# Questionnaire assessment helps the self-management of patients with inflammatory bowel disease during the outbreak of Coronavirus Disease 2019

**DOI:** 10.18632/aging.103525

**Published:** 2020-07-03

**Authors:** Meiping Yu, Zhenghao Ye, Yu Chen, Tingting Qin, Jiguang Kou, De’an Tian, Fang Xiao

**Affiliations:** 1 Department of Gastroenterology, Tongji Hospital, Tongji Medical College, Huazhong University of Science and Technology, Wuhan, China; 2 Department of Biliary–Pancreatic Surgery, Tongji Hospital, Tongji Medical College, Huazhong University of Science and Technology, Wuhan, China; 3 Department of Gastroenterology, Xiaogan Hospital Affiliated to Wuhan University of Science and Technology, Wuhan, China

**Keywords:** questionnaire, inflammatory bowel disease, COVID-19, self-management

## Abstract

Objective: This study aimed to assess the disease conditions of patients with inflammatory bowel disease (IBD) in Hubei Province during the outbreak of Coronavirus Disease 2019 (COVID-19) by questionnaire online and guide their self-management during this epidemic.

Results: A total of 102 eligible questionnaires were included. No patient we surveyed reported a diagnosis of COVID-19. Our result showed that 67.86% of patients with ulcerative colitis (UC) and 80.43% of patients with Crohn's disease (CD) were in remission, 85.29%of patients had a good quality of life. Part of the patients (21.57%) reported their disease conditions worsening. The reduction in physical exercise was a risk factor for worsening conditions (OR=17.593, p=0.009). Some patients reported an alteration of medication regimens during the epidemic.

Conclusions: The epidemic of COVID-19 might have a certain impact on many aspects of Hubei IBD patients within four weeks after the traffic control. Doctors could utilize the results from our questionnaire to guide IBD patients’ self-management.

Methods: A questionnaire was designed containing the Harvey-Bradshaw Index (HBI), the 6-point Mayo Score, the short inflammatory bowel disease questionnaire (SIBDQ) and distributed to Hubei IBD patients online within four weeks of traffic control after the outbreak, it also included questions about patients’ self-reported disease conditions and their epidemiological features of COVID-19.

## INTRODUCTION

In December 2019, an outbreak of COVID-19 caused by the severe acute respiratory syndrome coronavirus 2 (SARS-CoV-2) emerged in Wuhan, the capital of Hubei Province, China [1, 2]. On January 23, 2020, the Chinese government implemented traffic controls to prevent the spread of COVID-19 [3]. We distributed questionnaires to IBD patients from February 18th to 20th, which is approximately four weeks after traffic control. As of 20 February 2020, there had been 75,465 cases of COVID-19 confirmed in mainland China, including 62,662 cases in Hubei Province. The number of confirmed diagnoses in Hubei Province is 83.03% of that in China, accounting for the majority. At the same time, medical resources had also shifted more towards the diagnosis and treatment of COVID-19 in Hubei Province. These factors made routine medical treatment and follow-up of patients with chronic diseases inconvenient. Under these influences, it is essential for patients with chronic diseases to self-manage under the guidance of the doctor in this particular period. Self-management is the process by which patients participate in decision-making and self-care under the guidance of the doctors [4]. Patient’s effective self-management can relieve symptoms and control disease activity to a certain extent [5].

Inflammatory bowel disease (IBD) is a type of chronic idiopathic bowel disease, includes Crohn's disease (CD) and ulcerative colitis (UC). IBD patients have varying degrees of immune disorders [6] so that they may be considered as virus-susceptible. It is particularly crucial for IBD patients to know how to manage by themselves during the epidemic. Self-management requires monitoring of diseases by doctors first [7]. We monitored the patient's disease status through a questionnaire in our study. The content of the survey and the concept of the questionnaire design came from the patient-reported outcome (PRO). PRO is a visual report of the patient's treatment and disease management results, emphasizing the patient's self-evaluation and subjective perception [8]. PRO contains objective and subjective evaluation contents, which may include disease status, changes in functional status before and after the intervention, HRQoL and the patient's personal impressions [9–12].

To assess patients’ disease activity, HRQoL, and self-reported disease conditions, we design a verified 60-item questionnaire based on the concept of PRO. First of all, our questionnaire included the 6-point Mayo, HBI and SIBDQ. These indexes were objective quantitative indicators designed to obtain detailed knowledge of the disease activity and HRQoL of these IBD patients. Secondly, there were questions to understand patients’ subjective perceptions of disease conditions. Finally, this questionnaire also included questions about COVID-19 epidemiological features of these IBD patients. We gave feedback to these IBD patients and guided them to develop targeted self-management programs after obtaining the information through the questionnaire.

## RESULTS

### Demographic characteristics of study participants

A proximately 350 electronic questionnaires were distributed and a total of 111 were returned. There were 102 valid questionnaires, with an effective rate of 91.89%. The nine questionnaires excluded were due to some missing items. The median age of participants was 34 years (IQR, 27.25-42.25; range, 14-66), and 66.67% of participants were men. There were 56 (54.90%) patients with ulcerative colitis and 46 (45.10%) patients with Crohn's disease. Among all the participants in our survey, no one has reported infection with SARS-CoV-2; no one had symptoms related to COVID-19 or had a history of exposure. Table 1 shows the demographic data and disease-related variables for all participants who agreed and completed the survey.

**Table 1:** Baseline characteristics of the study population. (n=102).

**Characteristics**	**Value**
Age, Median (min-max) (IQR), y	34 (14-66) (27.25-42.25)
Gender	
Female	34 (33.33%)
Male	68 (66.67%)
Diagnosis	
Ulcerative Colitis	56 (54.90%)
Crohn's Disease	46 (45.10%)
Diagnosed with COVID-19	0
Huanan seafood market exposure	0
Signs and symptoms of COVID-19	0
Habitation*	
Wuhan	26 (25.49%)
Xiaogan	22(21.57%)
Jingzhou	12 (11.76%)
Suizhou	8 (7.83%)
Xiangyang	6 (5.88%)
Huangshi	6 (5.88%)
Huanggang	5 (4.90%)
Yichang	5 (4.90%)
Jingmen	4 (3.92%)
Xianning	2 (1.96%)
Xiantao	2 (1.96%)
Enshi Tujia and Miao Autonomous Prefecture	2(1.96%)
Tianmen	1 (0.98%)
Qianjiang	1 (0.98%)

*All participants are located in Hubei Province.

### Disease activity

We used the 6-point Mayo and HBI to score and grade disease activity in UC and CD patients respectively. The results were shown in Table 2. The median 6-point Mayo score of UC patients was 1 (IQR, 0-3; range, 0-6). Of the 56 UC patients, 38 (67.86%) UC patients were in remission, 4 (7.14%) patients had mild activity, 13 (23.21%) patients had moderate activity, and 1 (1.79%) patients had severe activity. The median HBI of CD patients was 2 (IQR, 1-4; range, 0-12). Of the 46 CD patients, 37 (80.43%) CD patients were in remission, 4 (8.70%) patients had mild activity, 5 (40.87%) patients had moderate activity, and no patients had severe activity. There was not a statistically significant difference in the proportion of the disease activity stage between UC and CD patients (p = 0.301).

**Table 2:** Evaluation of participants' IBD disease activity.

	**Participants with Ulcerative Colitis(n=56)**	**Participants with Crohn's Disease(n=46)**	P-value*
Index, Median (IQR) (range)	6-point Mayo,1(0-3)(0-6)	HBI, 2 (1-4) (0-12)	
Disease activity stage			0.301
Remission phase, n (%)	38 (67.86%)	37 (80.43%)	
Mild active phase, n (%)	4 (7.14%)	4 (8.70%)	
Moderate active phase, n (%)	13 (23.21%)	5 (10.87%)	
Severe active phase, n (%)	1 (1.79%)	0 (0%)	

HBI: Harvey-Bradshaw Index.

*Chi-square test was used to test whether there was a statistically significant difference in the proportion of the disease activity between UC and CD patients.

### Quality of life

The median SIBDQ of all participants was 59 (IQR, 52.25-63; range, 34-70). The median SIBDQ of UC patients was 60 (IQR, 54.75-64; range, 35-70), and the median SIBDQ of CD patients was 58 (IQR, 52-62.75; range, 34-69). Among all participants, 87 (85.29%) patients had the good health-related quality of life (HRQoL) (SIBDQ ≥ 50). There were 49 (87.50%) UC patients who had good HRQoL, compared with 38 (82.61%) CD patients who had good HRQoL (p=0.338) (Table 3), suggesting HRQoL is not significantly different between UC and CD patients.

**Table 3:** Evaluation of participants' SIBDQ.

	**Total participants (n=102)**	**Participants with UC and CD respectively**		
		**Participants with UC (n=56)**	**Participants with CD (n=46)**	P-value*
SIBDQ, Median (IQR)	59 (52.25-63)	60 (54.75-64)	58 (52-62.75)	
HRQoL				0.338
Good (≥ 50^1^), n (%)	87 (85.29%)	49 (87.50%)	38 (82.61%)	
Poor (<50), n (%)	15 (14.71%)	7 (15.50%)	8 (17.39%)	

HRQoL: health-related quality of life

1: SIBDQ score of more than 50 are considered to have a good HRQoL

*Chi-square test was used to test whether there was a statistically significant difference in the proportion of HRQoL between UC and CD patients.

### Self-reported disease conditions

In this questionnaire, we investigated the change of the patient's self-reported disease condition through the patient's subjective report. Approximately half of the patients (n = 55, 53.92%) thought that their disease condition did not change during the epidemic, 25 (24.51%) considered their disease condition improved, and 22 (21.57%) considered their disease condition worsening. We attempted to study the risk factors of change in patients' self-reported disease conditions. The result showed that reduced physical exercise was a risk factor for worse in the disease condition (OR=17.593, 95%CI 2.035 to 152.097, p=0.009). The other factors did not have a significant risk for change in the patient's disease condition. These data are shown in Table 4. The status of these factors during the epidemic came from the patients’ personal reports.

**Table 4:** Multi-variable logistic regression of the causes of change in participants’ self-evaluation disease condition.

**Disease condition**	**Characters**	**n (%)**	**OR(95%CI)**	**P-value**
Condition worsening (n=22)	Subsequent visit		
	No	18 (81.82%)	3.785(0.871, 16.458)	0.076
	Yes	4 (18.18%)		
		Medication regimen		
	Not changed	14 (63.64%)	0.264 (0.060, 1.167)	0.079
	Changed	8 (36.36%)		
		Emotional status		
	Negative	4 (18.18%)	3.306(0.413, 26.454)	0.260
	Positive	3 (13.64%)	0.830 (0.178, 3.880)	0.813
	Normal	15 (68.18%)		
		Physical exercise		
	Reduced	5 (22.73%)	17.593 (2.035, 152.097)	0.009
	Not reduced	17 (77.27%)		
		Rest		
	Inadequate	21 (95.45%)	1.071 (0.262, 4.378)	0.924
	Adequate	1 (4.55%)		
		Smoking		
	No	19 (86.36%)	0.665 (0.123, 3.591)	0.636
	Yes	3 (13.64%)		
Condition improved (n=25)		Subsequent visit		
	No	21 (84.00%)	2.730 (0.731, 10.199)	0.135
	Yes	4 (16.00%)		
		Medication regimen		
	Not changed	19 (76.00%)	0.374 (0.094, 1.496)	0.165
	Changed	6 (24.00%)		
		Emotional status		
	Negative	1 (4.00%)	2.094 (0.138, 31.710)	0.594
	Positive	14 (56.00%)	2.259 (0.751, 6.792)	0.147
	Normal	10 (40.00%)		
		Physical exercise		
	Reduced	1 (4.00%)	0.694 (0.222, 2.167)	0.529
	Not reduced	24 (96.00%)		
		Rest		
	9 (36.00%)	0.136 (0.015, 1.249)		0.078
	Adequate	16 (64.00%)		
		Smoking		
	Yes	4 (15.38%)	0.481 (0.104, 2.228)	0.350
	No	22 (84.62%)		

*The category with no change in the condition (n=55) was used as the reference category for the two groups of condition worsening (n=22) and condition improved (n=25).

### The change in medication regimen

We studied participants' medication regimens before and after the outbreak of COVID-19. Among UC patients, there was an increase of 3 patients who took no medication due to the discontinuation of mesalazine. The reason for the withdrawal of mesalazine was that the medicine was not available. Among CD patients, the number of patients who used adalimumab or took no medication increased, and the number of patients who used Remicade or methotrexate decreased. The details were shown in Table 5. The reasons for changing the medication regimens of these CD patients were “inability to purchase medication” and “inability to go to the hospital for routine treatment”. Among the reasons for these patients who changed their medication regimens, no one chose the options of “forgetting to take medicine” and “reducing medicine on your own”. This result represented that IBD patients' medical compliance during the epidemic was excellent in our survey.

**Table 5:** The comparison of the medication regimens before and after the outbreak of COVID-19 and the changes in medication regimens.

	**Medication**	**Before the outbreak, n (%)**	**After the outbreak, n (%)**	The number of changes*, n
	No medication	1 (1.75%)	4 (5.26%)	+3
	Mesalazine	50 (89.47%)	47 (85.96%)	-3
	Enteral nutrition	1 (1.75%)	1 (1.75%)	0
UC patients, (n=56)	Glucocorticoid	2 (3.51%)	2 (3.51%)	0
	Probiotics	4 (5.26%)	4 (5.26%)	0
	Biological therapy			
	Remicade	3 (5.26%)	3 (5.26%)	0
	Adalimumab	1 (1.75%)	1 (1.75%)	0
	No medication	1 (1.92%)	3 (5.77%)	+2
	Mesalazine	8 (17.31%)	8 (17.31%)	0
	Enteral nutrition	10 (19.23%)	10 (21.15%)	0
	Glucocorticoid	2 (3.85%)	2 (3.85%)	0
	Probiotics	1 (1.92%)	1 (1.92%)	0
CD patients, (n=46)	Biological therapy			
	Remicade	21 (46.15%)	16 (36.54%)	-5
	Adalimumab	1 (1.92%)	2 (3.85%)	+1
	Immunomodulators			
	Methotrexate	2 (3.85%)	1 (1.92%)	-1
	Azathioprine	20 (40.38%)	20 (40.38%)	0
	Thalidomide	1 (1.92%)	1 (1.92%)	0

*Plus sign indicates an increase in quantity and minus sign indicates a decrease in quantity.

### Emotional state

Negative emotions are significantly correlated with clinical recurrence and are also considered to be independent risk factors for more frequent relapse of disease [13, 14]. Therefore, we also investigated the emotional states of IBD patients in this survey. Changes in emotional states came from the patients' self-judgments. More than half of the participants (57.85%) thought they had the ordinary moods during this epidemic, 35.29% had positive moods, and 6.86% had negative moods.

## DISCUSSION

The COVID-19 epidemic outbreak emerged in Wuhan, Hubei Province of China in December 2019 [15]. The population was generally susceptible to SARS-CoV-2, especially for the elderly and the people with underlying diseases, who are prone to serious consequences [16, 17]. In our survey, there was no patient reported infection with SARS-CoV-2, but this did not mean that IBD patients were not susceptible to SARS-CoV-2 since our small sample size and the participants of this study were not obtained by randomized sampling. Until now, there is no evidence to prove the susceptibility of IBD patients to COVID-19 from other studies [18]. The epidemic led to the difficulty of maintaining previous disease management for IBD patients due to the contraction in routine medical resources. Our research focused on guiding the patient's self-management through the results of the questionnaire.

The traffic control in China from late January might largely change the lifestyle, psychological, and physical condition of the Chinese population [19–21], especially for residents in Hubei Province. We used the web questionnaire to evaluate the disease activity, HRQoL and the self-reported disease condition of IBD patients. Then we provided feedback on the patients' conditions and advised on their self-management. This manner helped IBD patients to adjust their treatment plans and develop a home-based self-management medical intervention model. Active doctor-patient communication can improve patients’ confidence with treatment, shared decision-making capacity and then provide a good impact on disease activity [4, 22]. We will continue to distribute questionnaires to this group of IBD patients in Hubei Province every month to guide patients’ home-based self-management during the epidemic of COVID-19.

Our results showed that 67.86% of UC patients and 80.43% of CD patients were in remission assessed by the 6-point Mayo score and HBI index. 85.29% of patients had a good HRQoL during the epidemic through the SIBDQ test. This suggested that more than half of the patients were in remission and had a good HRQoL in the early period of traffic control after the outbreak of COVID-19. With regard to the patients' self-reported results, although 78.43% of the patients thought that their disease conditions had not changed or even improved, there were still 21.57% of the patients considered that their disease conditions worsening. This showed that the early epidemic also had a certain impact on the patient's disease condition.

We studied the influencing factors of the self-reported disease condition of IBD patients during this epidemic. The factors included “reduction of exercise”, “emotional state”, “change of medication regimen”, “daily rest” and “subsequent visit”. The results showed that the reduction of exercise was a risk factor for worsening disease (OR=17.593, p=0.009). The other factors that could affect the disease conditions of IBD patients in previous studies [23–27] didn’t show a statistical correlation in our survey. This might be because of our small sample size. Therefore, we still recommend that IBD patients maintain a positive emotional state, retain the medication regimen, have adequate rest and make timely doctor-patient communication during the self-management process.

Pharmacological intervention is a key part of managing symptoms and maintaining remission in patients with IBD [28]. Our results showed that the number of people who took no medication increased among UC and CD patients after the outbreak of COVID-19. The number of UC patients taking mesalazine decreased due to the inconvenience of obtaining medications. For CD patients, the use of Remicade or methotrexate decreased because Remicade infusion in the hospital was not accessible and methotrexate could not be purchased. The use of adalimumab increased because we recommended patients to replace inaccessible Remicade with other available biologics. We tried to develop personalized treatment plans for specific patients on time to minimize the impact of changes in medication regimens on the patient's disease condition during the epidemic. It was an important step to increase the self-management of IBD patients.

The result showed that 93.14% of patients had normal or even positive emotional states, which implied that the epidemic did not have a very negative impact on the mood of these IBD patients in such a relatively early month. However, since previous studies have shown that emotional stress is significantly associated with decreased quality of life [29, 30], it was very important to intervene in the emotional state in the process of IBD patient self-management guidance.

### Limitations

The major limitation was that the sample size was small and our participants were not obtained by randomized sampling. In addition, there was no IBD patient diagnosed with COVID-19 in our study. Further researches need to be done to get more evidence about the susceptibility to SARS-CoV-2 in IBD patients and the clinical manifestations of IBD patients complicated with COVID-19.

## CONCLUSION

In the middle of February, although more than half of IBD patients we studied in Hubei Province were in remission and possessed a good HRQoL, the outbreak of COVID-19 still had a certain impact on IBD patients in Hubei Province, such as worsening of their self-reported disease conditions and changes in their treatment options. These changes deserved attention to the impact of epidemics on IBD patients. In our survey, doctors used the questionnaire to assess patients' disease conditions, give timely feedback and suggestions to IBD patients. This method facilitated the effective self-management of patients under the circumstance of the COVID-19 outbreak.

## MATERIALS AND METHODS

### Study design and participants

This study was an online questionnaire survey among IBD patients from the region of Hubei Province. From February 18, 2020 to February 20, 2020, the questionnaire was administered to a sample of IBD patients with regular follow-up in our IBD center. There were two items of the inclusion criteria for this study. One was that the patient (aging from 14 to 80) was established diagnosis of IBD for at least three months, another was that the patient is available to finish the online questionnaire (by Wechat, QQ, website, email) by himself or with the help of others. The exclusion criterion was that the patient is not able to finish the questionnaire. During this study, patients with IBD had been informed of the study’s aim. An IBD specialist nurse is explicitly trained in this questionnaire contacted them to explain the study objectives. Participants completed the questionnaire with an online survey portal. They completed questionnaires voluntarily and independently, under uncompensated conditions. This study was approved by the National Health Commission of China and Ethics Commission of Tongji Hospital, Tongji Medical College, Huazhong University of Science and Technology.

### Questionnaire design

The questionnaire was a validated 60-item questionnaire that assesses IBD disease activity across multiple domains, including the 6-point Mayo, HBI and SIBDQ (Supplementary File 1). HBI score ranges from 0 to 16 or more, and the highest score depends on the number of liquid stools per day. HBI scores of 0 and 4 are assigned to the remission phase; 5 to 7 are assigned to mildly active disease phase; 8 to 16 are assigned to moderately active disease phase; ≥17 are assigned to severely active disease phase [31, 32]. We used 6-point Mayo, which composed of the stool frequency, bleeding components. 6-point Mayo score of 0 and 1.5 are assigned to the remission phase; 1.5 to 2.5 are assigned to mildly active disease phase; 2.5 to 4.5 are assigned to moderately active disease phase; 8 to 4.5 are assigned to severely active disease phase [33, 34]. Total SIBDQ score ranges from 10 to 70, to find out how the patients have been feeling during the last two weeks. They will be asked about symptoms related to IBD diagnosis, as well as the general emotional status during the period and their attitude to the plague. Patients with a SIBDQ score of more than 50 are considered to have a good HRQoL [35–37].

In addition to the question about HBI, 6-point Mayo score and SIBDQ, the questionnaire included questions regarding the subjective feeling about their change in disease conditions and epidemiological history questions about COVID-19.

### Statistical analysis

Data collection and its statistical analysis were carried out using the SPSS software system (SPSS for Windows, Version 23.0, SPSS Inc., Chicago). Data analysis excluded incomplete items. Categorical data obtained were presented as frequency counts and percentages. Median, range and frequency were used to describe the demographic, SIBDQ scores and clinical characteristics. Chi-square tests were used to test the significant difference of categorical variables between two groups, such as the participants’ distribution in different disease active phases and HRQoL between UC and CD patients, with fisher exact test as appropriate. The logistic regression analysis was used to identify variables significantly associated with disease activity, and a multivariate model was built to assess the effect of each potential confounding factor and determined independent and significant factors associated with the disease index. The odds ratio (OR) was calculated to quantify the corresponding risk. For all analyses, p<0.05 was considered statistically significant.

## Supplementary Material

Supplementary File 1

## References

[1] Wang C, Horby PW, Hayden FG, Gao GF. A novel coronavirus outbreak of global health concern. Lancet. 2020; 395:470–73. 10.1016/S0140-6736(20)30185-9 31986257 PMC7135038

[2] The Lancet. Emerging understandings of 2019-nCoV. Lancet. 2020; 395:311. 10.1016/S0140-6736(20)30186-0 31986259 PMC7134625

[3] Chen S, Yang J, Yang W, Wang C, Bärnighausen T. COVID-19 control in China during mass population movements at New Year. Lancet. 2020; 395:764–66. 10.1016/S0140-6736(20)30421-9 32105609 PMC7159085

[4] Kennedy A, Gask L, Rogers A. Training professionals to engage with and promote self-management. Health Educ Res. 2005; 20:567–78. 10.1093/her/cyh018 15741189

[5] Osman L. Guided self-management and patient education in asthma. Br J Nurs. 1996; 5:785–89. 10.12968/bjon.1996.5.13.785 8974523

[6] Bouma G, Strober W. The immunological and genetic basis of inflammatory bowel disease. Nat Rev Immunol. 2003; 3:521–33. 10.1038/nri1132 12876555

[7] Robinson A, Thompson DG, Wilkin D, Roberts C, and Northwest Gastrointestinal Research Group. Guided self-management and patient-directed follow-up of ulcerative colitis: a randomised trial. Lancet. 2001; 358:976–81. 10.1016/S0140-6736(01)06105-0 11583752

[8] Breitscheidel L, Stamenitis S. Using patient-reported outcome assessments in clinical practice and their importance in risk management. J Med Econ. 2009; 12:180–81. 10.3111/13696990903216278 19691445

[9] Bradley C. Feedback on the FDA's February 2006 draft guidance on Patient Reported Outcome (PRO) measures from a developer of PRO measures. Health Qual Life Outcomes. 2006; 4:78. 10.1186/1477-7525-4-78 17029628 PMC1634851

[10] Black N, Jenkinson C. Measuring patients’ experiences and outcomes. BMJ. 2009; 339:b2495. 10.1136/bmj.b2495 19574317

[11] Elkjaer M, Shuhaibar M, Burisch J, Bailey Y, Scherfig H, Laugesen B, Avnstrøm S, Langholz E, O’Morain C, Lynge E, Munkholm P. E-health empowers patients with ulcerative colitis: a randomised controlled trial of the web-guided ‘Constant-care’ approach. Gut. 2010; 59:1652–61. 10.1136/gut.2010.220160 21071584

[12] Acquadro C, Berzon R, Dubois D, Leidy NK, Marquis P, Revicki D, Rothman M, and PRO Harmonization Group. Incorporating the patient's perspective into drug development and communication: an ad hoc task force report of the Patient-Reported Outcomes (PRO) Harmonization Group meeting at the Food and Drug Administration, February 16, 2001. Value Health. 2003; 6:522–31. 10.1046/j.1524-4733.2003.65309.x 14627058

[13] Keefer L, Kiebles JL, Taft TH. The role of self-efficacy in inflammatory bowel disease management: preliminary validation of a disease-specific measure. Inflamm Bowel Dis. 2011; 17:614–20. 10.1002/ibd.21314 20848516 PMC3005084

[14] Mikocka-Walus A, Knowles SR, Keefer L, Graff L. Controversies Revisited: A Systematic Review of the Comorbidity of Depression and Anxiety with Inflammatory Bowel Diseases. Inflamm Bowel Dis. 2016; 22:752–62. 10.1097/MIB.0000000000000620 26841224

[15] Sohrabi C, Alsafi Z, O’Neill N, Khan M, Kerwan A, Al-Jabir A, Iosifidis C, Agha R. World Health Organization declares global emergency: A review of the 2019 novel coronavirus (COVID-19). Int J Surg. 2020; 76:71–76. 10.1016/j.ijsu.2020.02.034 32112977 PMC7105032

[16] Guo YR, Cao QD, Hong ZS, Tan YY, Chen SD, Jin HJ, Tan KS, Wang DY, Yan Y. The origin, transmission and clinical therapies on coronavirus disease 2019 (COVID-19) outbreak - an update on the status. Mil Med Res. 2020; 7:11. 10.1186/s40779-020-00240-0 32169119 PMC7068984

[17] Tian S, Hu N, Lou J, Chen K, Kang X, Xiang Z, Chen H, Wang D, Liu N, Liu D, Chen G, Zhang Y, Li D, et al. Characteristics of COVID-19 infection in Beijing. J Infect. 2020; 80:401–06. 10.1016/j.jinf.2020.02.018 32112886 PMC7102527

[18] Mao R, Liang J, Shen J, Ghosh S, Zhu LR, Yang H, Wu KC, Chen MH, and Chinese Society of IBD, Chinese Elite IBD Union, Chinese IBD Quality Care Evaluation Center Committee. Implications of COVID-19 for patients with pre-existing digestive diseases. Lancet Gastroenterol Hepatol. 2020; 5:425–27. 10.1016/S2468-1253(20)30076-5 32171057 PMC7103943

[19] Shigemura J, Ursano RJ, Morganstein JC, Kurosawa M, Benedek DM. Public responses to the novel 2019 coronavirus (2019-nCoV) in Japan: mental health consequences and target populations. Psychiatry Clin Neurosci. 2020; 74:281–82. 10.1111/pcn.12988 32034840 PMC7168047

[20] Wang C, Pan R, Wan X, Tan Y, Xu L, Ho CS, Ho RC. Immediate Psychological Responses and Associated Factors during the Initial Stage of the 2019 Coronavirus Disease (COVID-19) Epidemic among the General Population in China. Int J Environ Res Public Health. 2020; 17:E1729. 10.3390/ijerph17051729 32155789 PMC7084952

[21] Allam Z, Jones DS. On the Coronavirus (COVID-19) Outbreak and the Smart City Network: Universal Data Sharing Standards Coupled with Artificial Intelligence (AI) to Benefit Urban Health Monitoring and Management. Healthcare (Basel). 2020; 8:E46. 10.3390/healthcare8010046 32120822 PMC7151018

[22] Barlow C, Cooke D, Mulligan K, Beck E, Newman S. A critical review of self-management and educational interventions in inflammatory bowel disease. Gastroenterol Nurs. 2010; 33:11–8. 10.1097/SGA.0b013e3181ca03cc 20145446

[23] Boye B, Lundin KE, Jantschek G, Leganger S, Mokleby K, Tangen T, Jantschek I, Pripp AH, Wojniusz S, Dahlstroem A, Rivenes AC, Benninghoven D, Hausken T, et al. INSPIRE study: does stress management improve the course of inflammatory bowel disease and disease-specific quality of life in distressed patients with ulcerative colitis or Crohn’s disease? A randomized controlled trial. Inflamm Bowel Dis. 2011; 17:1863–73. 10.1002/ibd.21575 21287660

[24] Ranjbaran Z, Keefer L, Stepanski E, Farhadi A, Keshavarzian A. The relevance of sleep abnormalities to chronic inflammatory conditions. Inflamm Res. 2007; 56:51–7. 10.1007/s00011-006-6067-1 17431741

[25] Narula N, Fedorak RN. Exercise and inflammatory bowel disease. Can J Gastroenterol. 2008; 22:497–504. 10.1155/2008/785953 18478136 PMC2660805

[26] Ng V, Millard W, Lebrun C, Howard J. Low-intensity exercise improves quality of life in patients with Crohn's disease. Clin J Sport Med. 2007; 17:384–88. 10.1097/JSM.0b013e31802b4fda 17873551

[27] Lenti MV, Selinger CP. Medication non-adherence in adult patients affected by inflammatory bowel disease: a critical review and update of the determining factors, consequences and possible interventions. Expert Rev Gastroenterol Hepatol. 2017; 11:215–26. 10.1080/17474124.2017.1284587 28099821

[28] Lamb CA, Kennedy NA, Raine T, Hendy PA, Smith PJ, Limdi JK, Hayee B, Lomer MC, Parkes GC, Selinger C, Barrett KJ, Davies RJ, Bennett C, et al, and IBD guidelines eDelphi consensus group. British Society of Gastroenterology consensus guidelines on the management of inflammatory bowel disease in adults. Gut. 2019 (Suppl 3); 68:s1–106. 10.1136/gutjnl-2019-318484 31562236 PMC6872448

[29] Slonim-Nevo V, Sarid O, Friger M, Schwartz D, Chernin E, Shahar I, Sergienko R, Vardi H, Rosenthal A, Mushkalo A, Dizengof V, Ben-Yakov G, Abu-Freha N, et al, and Israeli IBD Research Nucleus (IIRN). Effect of psychosocial stressors on patients with Crohn’s disease: threatening life experiences and family relations. Eur J Gastroenterol Hepatol. 2016; 28:1073–81. 10.1097/MEG.0000000000000666 27203602

[30] Slonim-Nevo V, Sarid O, Friger M, Schwartz D, Sergienko R, Pereg A, Vardi H, Singer T, Chernin E, Greenberg D, Odes S, Dotan I, Chowers Y, et al, and Israeli IBD Research Nucleus (IIRN). Effect of Social Support on Psychological Distress and Disease Activity in Inflammatory Bowel Disease Patients. Inflamm Bowel Dis. 2018; 24:1389–400. 10.1093/ibd/izy041 29893949

[31] Greenberg D, Schwartz D, Vardi H, Friger M, Sarid O, Slonim-Nevo V, Odes S, and Israeli IBD Research Nucleus [IIRN]. Health-Related Utility Weights in a Cohort of Real-World Crohn’s Disease Patients. J Crohn’s Colitis. 2015; 9:1138–45. 10.1093/ecco-jcc/jjv167 26374662

[32] Hüppe A, Langbrandtner J, Häuser W, Raspe H, Bokemeyer B. Validation of the “German Inflammatory Bowel Disease Activity Index (GIBDI)”: An Instrument for Patient-Based Disease Activity Assessment in Crohn’s Disease and Ulcerative Colitis. Z Gastroenterol. 2018; 56:1267–75. 10.1055/a-0605-4080 29742780

[33] Bewtra M, Brensinger CM, Tomov VT, Hoang TB, Sokach CE, Siegel CA, Lewis JD. An optimized patient-reported ulcerative colitis disease activity measure derived from the Mayo score and the simple clinical colitis activity index. Inflamm Bowel Dis. 2014; 20:1070–78. 10.1097/MIB.0000000000000053 24810138 PMC4137887

[34] de Jong MJ, Huibregtse R, Masclee AAM, Jonkers DMAE, Pierik MJ. Patient-Reported Outcome Measures for Use in Clinical Trials and Clinical Practice in Inflammatory Bowel Diseases: A Systematic Review. Clin Gastroenterol Hepatol. 2018; 16:648–663.e3. 10.1016/j.cgh.2017.10.019 29074448

[35] Gonczi L, Kurti Z, Verdon C, Reinglas J, Kohen R, Morin I, Chavez K, Bessissow T, Afif W, Wild G, Seidman E, Bitton A, Lakatos PL. Perceived Quality of Care is Associated with Disease Activity, Quality of Life, Work Productivity, and Gender, but not Disease Phenotype: A Prospective Study in a High-volume IBD Centre. J Crohn’s Colitis. 2019; 13:1138–47. 10.1093/ecco-jcc/jjz035 30793162

[36] Hlavaty T, Persoons P, Vermeire S, Ferrante M, Pierik M, Van Assche G, Rutgeerts P. Evaluation of short-term responsiveness and cutoff values of inflammatory bowel disease questionnaire in Crohn’s disease. Inflamm Bowel Dis. 2006; 12:199–204. 10.1097/01.MIB.0000217768.75519.32 16534421

[37] Ananthakrishnan AN, Weber LR, Knox JF, Skaros S, Emmons J, Lundeen S, Issa M, Otterson MF, Binion DG. Permanent work disability in Crohn’s disease. Am J Gastroenterol. 2008; 103:154–61. 10.1111/j.1572-0241.2007.01561.x 18076736

