# Supplementary material for: Questionnaire assessment helps the self-management of patients with inflammatory bowel disease during the outbreak of Coronavirus Disease 2019
Source: Aging (Albany NY). 2020 Jul 3;12(13):12468–78. doi: 10.18632/aging.103525 (PMC7377832; doi:10.18632/aging.103525)
Supplement: Supplementary File 1 [file aging-12-103525-s001..docx]

**Follow-up of patients with inflammatory bowel disease during the outbreak of Corona Virus Disease 2019**

1. Your name: ______
2. Your gender:

□Male

□Female

1. Your age: ______
2. Your location: (province, city)
3. Your diagnosis:

□Ulcerative colitis

□Crohn's disease

1. Are you infected with SARS-CoV-2?

□Yes

□No

1. Have you ever been to Huanan seafood market since the outbreak of COVID-19?

□Yes

□No

1. Have you been in contact with patients with [diagnose](https://www.baidu.com/link?url=isiu5fT4hcSohFuWUsiCcS2UUmxSYldmdik4TerkPj9yYkmOLB0P8BFI7pGWsmRh1vVVGCfvYkrK8cL8mXuvLeezwhsFGxKN4hNKrLPeY5O&wd=&eqid=8b1f6ed90009f376000000025e71f256)d COVID-19?

□Yes

□No

1. Do you have any relatives infected with SARS-CoV-2?

□Yes

□No

1. Is someone in your community infected with SARS-CoV-2?

□Yes

□No

1. Have you ever been exposed to wild animals?

□Yes

□No

1. What is your current status?

□Isolate in the home

□Isolate in the official isolation zone

□Hospitalization

1. How many times have you been away from home every week since the outbreak of COVID-19?

□Go out once a week

□Go out 2-3 times a week

□Go out 4-5 times a week

□Go out > 5 times a week

1. Have you had fever, cough, vomiting and other symptoms in the past four weeks

□Yes__________

□No

1. Do you wear a mask to protect yourself when you go out?

□Every time

□Most of the time

□Occasionally

□Never

1. Do you wash your hands frequently and pay attention to personal hygiene every day

□Yes

□No

1. What do you think of the symptoms of your inflammatory bowel disease compared to before the outbreak of Corona Virus Disease 2019 (COVID-19)

□improved

□same as before

□worsening

1. Did you get adequate rest during the period of COVID-19?

□Adequate

□Inadequate

1. Did you reduce your physical exercise during the period of COVID-19?

□Reduced

□Not reduced

□Not change

1. Did your intake of fresh fruits and vegetables change during the period of COVID-19?

□Decrease

□No change

□Increase

1. Did your intake of meat, eggs and dairy products change during the period of COVID-19?

□Decrease

□No change

□Increase

1. Do you smoke

□Yes

□No

1. How many cigarettes do you smoke per week?

□ ≤1

□ 2-5

□ 6-10

□ 10 or more

1. How about the character of your stool recently

□Normal

□Soft stool

□Diarrhea

1. Please choose an option to describe your general well-being

□very well

□slightly below par

□poor

□very poor

□terrible

1. Please choose an option to describe your abdominal pain

□none

□mild

□moderate

□severe

1. Number of loose or watery stools in the last week

Monday___________

Tuesday___________

Wednesday___________

Thursday___________

Friday___________

Saturday___________

Sunday___________

1. Please choose an option to describe your abdominal mass

□ none

□dubious

□definite

□ definite and tender

1. Do you have the following complications?
2. Arthralgia / Arthritis

□yes □no

1. Uveitis / Iriditis

□yes □no

1. Erythema nodosum

□yes □no

1. Aphthous ulcer

□yes □no

1. Pyoderma gangrenosum

□yes □no

1. Anal fissure

□yes □no

1. New fistula

□yes □no

1. Abscess

□yes □no

1. Please select an option to describe your current bleeding condition

□None

□Visible blood with stool less than half the time

□Visible blood with stool half of the time or more

□Passing blood alone

1. Do you need to have a subsequent visit or see a doctor during the epidemic?

□Yes

□No

1. Have you had any subsequent visits since the outbreak of COVID-19?

□Yes

□No

1. The reason why you did not have any subsequent visit is

□Worried about infecting SARS-CoV-2 and not went to the hospital on time

□Traffic Control

□Outpatient service is closed during the epidemic period

□Not yet time for the subsequent visit

□Forget the subsequent visit time

□Other reasons___________________

1. Did you go to the doctor conveniently during the epidemic?

□Conveniently

□Some affected

□Very affected

□Couldn't seek medical treatment at all

1. How did you choose to see a doctor during the epidemic?

□Go to the hospital

□Online medical treatment

□Go to the clinic or community hospital

□No medical treatment

1. When was your last time for [hematologic examination](http://www.baidu.com/link?url=gF2MpXICsDszdJGRTLn6zoXKGvjff4jMw74ME-vnfdr8HoQlRCruhxjzAw3tU7Ac3BnDEhIa8i1KDt56LTo2VtptvzkXzj0DoNdsXZeifq8p6xKL2UdeOFJsPR4G-v7t)? (not required)

_______________________________________________________

1. The result of your last [hematologic examination](http://www.baidu.com/link?url=gF2MpXICsDszdJGRTLn6zoXKGvjff4jMw74ME-vnfdr8HoQlRCruhxjzAw3tU7Ac3BnDEhIa8i1KDt56LTo2VtptvzkXzj0DoNdsXZeifq8p6xKL2UdeOFJsPR4G-v7t) is (not required)

_______________________________________________________

1. When was your last subsequent visit (not required)

_______________________________________________________

1. What examinations were carried out in the last subsequent visit and what were the results? (not required)

_______________________________________________________

1. What was your medication regimen for inflammatory bowel disease before the outbreak of COVID-19?

□Mesalazine

□Glucocorticoid

□Azathioprine

□Thalidomide

□Remicade

□Adalimumab

□Enteral nutrition

□Other medications____________________

1. Did you change your medication regimen for inflammatory bowel disease during the period of COVID-19?

□No change

□Change

1. If you changed your medication regimen, what is your medication regimen for inflammatory bowel disease during the period of COVID-19?

□Mesalazine

□Glucocorticoid

□Azathioprine

□Thalidomide

□Remicade

□Adalimumab

□Enteral nutrition

□Other medications_____________________

1. The reason why you changed your medication regimen

□Unable to purchase and obtain drugs

□Forgetting to take medicine

□Reducing medicine on your own

□□Reducing medicine according to the doctor's advice

□Other______________________

1. How was your emotional state during the period of COVID-19?

□Positive

□General

□Negative

1. Are you anxious about the outbreak of COVID-19

□Yes

□No

1. Do you think whether the emotion changes had effects on the symptoms of your inflammatory bowel disease

□Positive Impact

□No impact

□Negative Impact

1. How often has the feeling of fatigue or of being tired and worn out been a problem for you during the last 2 wk? Please choose an option from

□All of the time

□Most of the time

□A good bit of the time

□Some of the time

□A little of the time

□Hardly any of the time

□None of the time

1. How often during the last 2 wk have you had to delay or cancel a social engagement because of your bowel problem? Please choose an option from

□All of the time

□Most of the time

□A good bit of the time

□Some of the time

□A little of the time

□Hardly any of the time

□None of the time

1. How much difficulty have you had, as a result of your bowel problems, doing leisure or sports activities you would have liked to have done over the last 2 wk? Please choose an option from

□A great deal of difficulty

□A lot of difficulties

□A fair bit of difficulty

□Some difficulty

□A little difficulty

□Hardly any difficulty

□No difficulty

1. How often during the last 2 wk have you been troubled by pain in the abdomen? Please choose an option from

□All of the time

□Most of the time

□A good bit of the time

□Some of the time

□A little of the time

□Hardly any of the time

□None of the time

1. How often during the last 2 wk have you felt depressed or discouraged? Please choose an option from

□All of the time

□Most of the time

□A good bit of the time

□Some of the time

□A little of the time

□Hardly any of the time

□None of the time

1. Overall, in the last 2 wk, how much of a problem have you had passing large amounts of gas? Please choose an option from

□A major problem

□A big problem

□A significant problem

□Some trouble

□A little trouble

□Hardly any trouble

□No trouble

1. Overall, in the last 2 wk, how much of a problem have you had maintaining or getting to the weight you would like to be? Please choose an option from

□A major problem

□A big problem

□A significant problem

□Some trouble

□A little trouble

□Hardly any trouble

□No trouble

1. How often during the last 2 wk have you felt relaxed and free of tension? Please choose an option from

□All of the time

□Most of the time

□A good bit of the time

□Some of the time

□A little of the time

□Hardly any of the time

□None of the time

1. How much of the time during the last 2 wk have you been troubled by a feeling of having to go to the toilet even though your bowels were empty? Please choose an option from

□All of the time

□Most of the time

□A good bit of the time

□Some of the time

□A little of the time

□Hardly any of the time

□None of the time

1. How much of the time during the last 2 wk have you felt angry as a result of your bowel problem? Please choose an option from

□All of the time

□Most of the time

□A good bit of the time

□Some of the time

□A little of the time

□Hardly any of the time

□None of the time

1. Do you have other chronic diseases besides inflammatory bowel disease (such as hypertension, chronic obstructive pneumonia, etc.)

□Yes

□No

1. What is your chronic disease?

□Malignant tumor

□Diabetes

□Hypertension

□Rheumatism

□Chronic viral hepatitis

□Obstructive emphysema

□Chronic Heart Failure

□Primary cardiomyopathy

□Coronary heart disease

□Chronic Glomerulonephritis

□Nephrotic Syndrome

□Other_____________

1. How has the chronic disease been maintained since the outbreak of COVID-19?

□Better than before

□Same as before

□Worse than before

1. Do you take other medications apart from the treatment of inflammatory bowel disease?

________________________________________________________________________________________________________________________________________________________________________________________________________________________________________________
